# Bridging Reinforcement Learning and Iterative Learning Control: Autonomous Motion Learning for Unknown, Nonlinear Dynamics

**DOI:** 10.3389/frobt.2022.793512

**Published:** 2022-07-12

**Authors:** Michael Meindl, Dustin Lehmann, Thomas Seel

**Affiliations:** ^1^ Embedded Mechatronics Laboratory, Hochschule Karlsruhe, Karlsruhe, Germany; ^2^ Department Artificial Intelligence in Biomedical Engineering, Friedrich-Alexander-Universität Erlangen-Nürnberg, Erlangen, Germany; ^3^ Control Systems Group, Technische Universität Berlin, Berlin, Germany

**Keywords:** autonomous systems, Gaussian processes (GP), iterative learning control, nonlinear systems, reinforcement learning, robot learning

## Abstract

This work addresses the problem of reference tracking in autonomously learning robots with unknown, nonlinear dynamics. Existing solutions require model information or extensive parameter tuning, and have rarely been validated in real-world experiments. We propose a learning control scheme that learns to approximate the unknown dynamics by a Gaussian Process (GP), which is used to optimize and apply a feedforward control input on each trial. Unlike existing approaches, the proposed method neither requires knowledge of the system states and their dynamics nor knowledge of an effective feedback control structure. All algorithm parameters are chosen automatically, i.e. the learning method works plug and play. The proposed method is validated in extensive simulations and real-world experiments. In contrast to most existing work, we study learning dynamics for more than one motion task as well as the robustness of performance across a large range of learning parameters. The method’s plug and play applicability is demonstrated by experiments with a balancing robot, in which the proposed method rapidly learns to track the desired output. Due to its model-agnostic and plug and play properties, the proposed method is expected to have high potential for application to a large class of reference tracking problems in systems with unknown, nonlinear dynamics.

## 1 Introduction

Recent developments in robotic technology remarkably contribute to the quality of human live: Hazardous tasks on rescue missions are handled by mobile robots that rifle through wreckage to locate people in need of help (Murphy, 2004). Advances in medical robotics strive for minimizing complications during surgery (Coulson et al., 2008). And the combination of exoskeletons and control algorithms aims for a future in which people struck by disability can walk again (Harib et al., 2018). The way to such accomplishments is paved by control techniques that enable robots to precisely perform agile and dynamic motions.

For example, model predictive control can achieve accurate motion if a precise model of the dynamics is available (Apgar et al., 2018; Hehn and D’Andrea, 2012; Feng et al., 2014). Requirements regarding the model’s precision can be relaxed by robust or adaptive control techniques if the uncertainties comply with preset assumptions (Dong and Kuhnert, 2005; Dydek et al., 2013; Golovin and Palis, 2019). Under similar conditions, Iterative Learning Control (ILC) can overcome model uncertainties and unknown disturbances by learning from errors of previous trials (Muller et al., 2012; Seel et al., 2016). However, all of these control approaches require system-specific prior knowledge to craft a suited model, controller, or learning configuration. In contrast, autonomy requires a methodology that self-reliantly learns a solution to the control problem without requiring any system-specific prior knowledge. In particular, Reinforcement Learning (RL) techniques have been employed to solve complex motion tasks without requiring any prior information. However, RL solutions typically suffer from two major drawbacks: First, the vast majority of the results were obtained in simulated environments (Heess et al., 2017; Tassa et al., 2018; Tsounis et al., 2020). Second, the few results obtained in real-world environments required at least multiple hours of learning, and the resulting controllers can be prone to failure (Schuitema, 2012; Kalashnikov et al., 2018; Ha et al., 2020; Zeng et al., 2020). The only exception from this statement is given by RL methods that exploit task-specific knowledge in the form of good initial policies and require 60–300 trials for local policy optimization (Kober and Peters, 2008; Peters and Schaal, 2008; Kormushev et al., 2013). A major breakthrough with respect to robustness and data-efficiency was achieved by hybrid techniques that learn parameter-free models, namely Gaussian Processes (GP), but also employ system-specific information such as knowledge of a state vector and an effective state feedback structure (Deisenroth, 2010). In the prominent example of *PILCO* (Deisenroth and Rasmussen, 2011a), experimental data are used to approximate the unknown dynamics by a GP, which is used to determine the optimal parameters of a state feedback controller. By this approach, an inverted pendulum on a cart could be swung up and stabilized in 12 s of system interaction (Deisenroth and Rasmussen, 2011a). However, in the context of autonomous motion learning, GP-based learning methods still suffer from two drawbacks: First, previously proposed methods only solve set-point stabilization tasks, which do not enable robots to perform challenging, dynamic maneuvers. These require reference tracking. Second, GP-based learning techniques still require system-specific prior knowledge such as the configuration of cost functions, a state vector that fully describes the system dynamics, and a control structure that is effective with respect to the problem at hand. Hence, the methods are not suited for plug and play learning of highly dynamic robotic motions.

**FIGURE 1:**
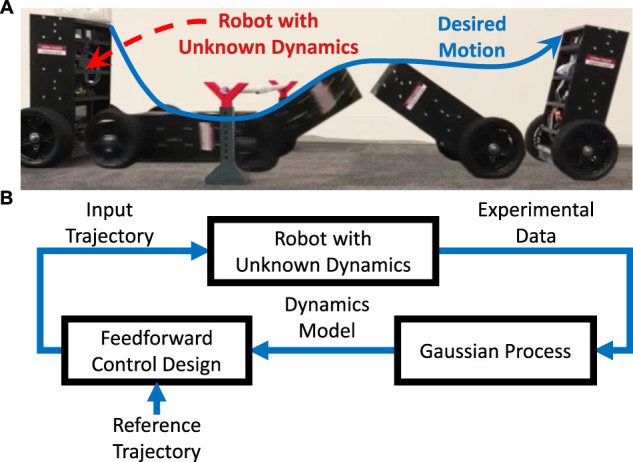
**(A)** A robot with unknown dynamics is meant to track a reference trajectory leading to a desired, highly dynamic motion. **(B)** On each iteration, the proposed learning method determines, based on experimental data, a Gaussian Process model, which is in turn used to design and apply a feedforward control input.

The present contribution proposes a GP-based learning method for autonomously solving highly dynamic reference tracking tasks in systems with unknown, nonlinear, single-input/single-output dynamics. The proposed method autonomously determines all of its necessary parameters such that plug and play application becomes feasible. The method’s capability to rapidly learn solutions to various reference tracking tasks while not requiring any system-specific prior knowledge is validated by extensive simulations and real-world experiments using a two-wheeled inverted pendulum robot, see Figure 1.

### 1.1 Related Work

**FIGURE 2:**
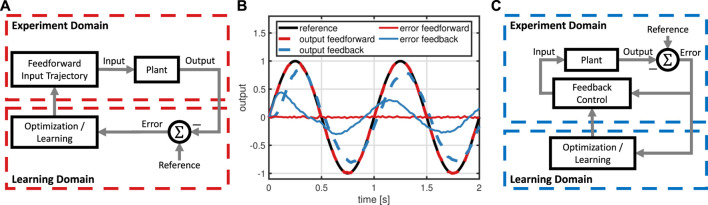
Comparison of feedforward **(A)** and feedback learning control **(C)** for reference tracking with a system affected by input delay and measurement noise: Feedforward, unlike feedback control, achieves almost perfect tracking **(B)**. Hence, the learning method proposed in this work employs feedforward control.

Learning for control has been considered in a large body of literature that can be categorized by 1) the considered control problem respectively control strategy, 2) necessary system-specific prior knowledge, and 3) speed of learning. Reinforcement Learning (RL) techniques typically do not require any model and only few learning parameters such as step sizes or weights in cost functions. Furthermore, general RL approaches such as genetic algorithms (Moriarty and Miikkulainen, 2007) or policy gradient approaches (Peters and Schaal, 2006) can be applied to arbitrary control problems with unknown, nonlinear dynamics, but in turn require comparatively long periods of learning (Deisenroth and Rasmussen, 2011a). The speed of learning can be significantly increased if the technique is targeted towards a specific control problem and strategy such as stabilization by state feedback control, see e.g. (Lewis and Vrabie, 2009; Lewis and Vamvoudakis, 2011). A particularly data-efficient approach are so called model-based techniques that model the unknown, nonlinear dynamics by a GP, which is then used to design a state feedback controller (Deisenroth, 2011). Some successful applications to real-world examples are the control of a single inverted pendulum (Deisenroth and Rasmussen, 2011a), double inverted pendulum (Hesse et al., 2018), and robotic manipulator (Deisenroth et al., 2012). The concept of GP-based learning control has been further investigated in a variety of contributions. Stability of feedback-controlled GPs has been analyzed (Vinogradska et al., 2017, 2016), the problem of computational and data requirements has been investigated (Nguyen-Tuong and Peters, 2008; Capone et al., 2020), and solutions for safely improving an existing feedback controller have been proposed (Berkenkamp and Schoellig, 2015; Hewing et al., 2017; Umlauft et al., 2020). In a similar fashion, a type of lazy learning methods constructs locally weighted models based on experimental data to design feedback controllers for set-point stabilization, see (Atkeson et al., 1997), but require problem-specific knowledge like, e.g., the configuration of cost functions. While all of these works consider the challenging problem of efficiently learning control solutions for unknown, respectively uncertain, nonlinear dynamics, they have focused on the problem of set-point stabilization of systems, for which an effective feedback control structure is known. If the control tasks consists in performing a highly-dynamic motion, the achievable performance of time-domain feedback control is inherently limited by phenomena such as unknown delays, measurement noise, or non-minimum phase dynamics. To overcome the performance limitations of feedback control, a feedforward control component is required, see Figure 2.

In contrast to GP-based learning techniques, Iterative Learning Control (ILC) has focused on reference tracking tasks solved by feedforward control (Ahn et al., 2007; Arimoto et al., 1984). Model-based techniques like norm-optimal or $H_{\infty }$ ILC automatically determine the learning parameters, but require a model of the linear plant dynamics (Tayebi and Zaremba, 2002; Gunnarsson and Norrlöf, 2001; Amann et al., 1996). Model-free approaches like PD-ILC do not require a model but learning parameters that are typically tuned in experiment (Bristow et al., 2005). The concepts of PD-type (Shen et al., 2016) and norm-optimal (Lu et al., 2018) ILC have been extended to the case of nonlinear dynamics, but assume the dynamics to be known. To relax requirements with respect to available model information, recent research has focused on so called data-driven ILC (DD-ILC) (Hou and Wang, 2013), which does not require a model of the plant. In the case of nonlinear, unknown dynamics, DD-ILC methods typically employ dynamic linearization of the plant dynamics and estimate the gradient of said linearization (Chi et al., 2015a,b; Ai et al., 2020). Alternatively, neural networks (NN) have been employed in DD-ILC to model the unknown dynamics (Ma et al., 2020; Yu et al., 2020). In a similar fashion, (Petric et al., 2018) propose basis functions for computing the input trajectory to track a desired reference, whereby the weights of the basis functions are learned by weighted linear regression, but the learning requires multiple hand-tuned parameters including the number of basis functions, step-sizes, and damping constants. In summary, while existing DD-ILC and similar methods can solve reference tracking tasks without requiring a plant model, some system-specific prior knowledge is required as, e.g., the signs of the dynamic linearization (Chi et al., 2015a,b; Ai et al., 2020), the layout of a suited neural network (Ma et al., 2020; Yu et al., 2020), or weights and step-sizes in update laws (Petric et al., 2018).

In summary, we conclude that reference tracking tasks in systems with unknown, nonlinear dynamics can be solved by DD-ILC methods, which, however, require system-specific prior knowledge such that autonomous plug and play application is generally not possible. In contrast, set-point stabilization problems can be solved by GP-based learning methods that assume comparatively little system-specific prior knowledge. However, in the context of reference tracking tasks, GP-based learning methods suffer from the inherent limitations of feedback control. To the best of our knowledge, there exists no learning method that autonomously solves reference tracking tasks for unknown, nonlinear systems, employs feedforward control to overcome the limitations of feedback control, and does not require system-specific prior knowledge such that autonomous plug and play application is enabled.

### 1.2 Contributions

In this contribution, a GP-based ILC scheme is proposed that autonomously solves reference tracking tasks in systems with unknown, nonlinear, single-input/single-output dynamics. The proposed method includes a procedure to autonomously determine necessary parameters and enable plug and play application. Since the method directly models the input/output dynamics, only the output variable, instead of an entire state vector, has to be known and measured. To overcome the inherent limitations of feedback control, the proposed method employs feedforward control.

The proposed method is first validated by extensive simulations of a two-wheeled inverted pendulum robot (TWIPR), in which precise tracking is achieved after a small number of trials. Unlike existing approaches, the proposed method is not only verified for a single, well-chosen parameter configuration but for a wide range of parameter combinations such that robustness with respect to the autonomously determined parameters is ensured. In contrast to a variety of contributions, in which validation was restricted to simulated environments, the proposed method’s capability of solving real-world reference tracking tasks in a plug and play manor is validated by experiments on a TWIPR, see Figure 1.

## 2 Problem Formulation

Consider an autonomous system that can repeatedly attempt a reference tracking task, as, e.g., a robot trying to perform a desired maneuver. We assume that the system’s output, e.g., a joint angle or position, can be influenced by an input signal, e.g., a motor torque, and that the relation of these variables is deterministic, causal, and time-invariant. However, we do *not* assume that a model of the dynamics is available and we *do* assume the general case of nonlinear dynamics.

Formally, consider a discrete-time, single-input, single-output, repetitive system with a finite trial duration of N∈N samples, and, on trial $j\in N_{\geq 0}$ and sample *n* ∈ [1, *N*], output variable $y_{j}\left(n\right)\in R$ , respectively input variable $u_{j}\left(n\right)\in R$ . The samples are collected in the so called output trajectory $y_{j}\in R^{N}$ , respectively input trajectory $u_{j}\in R^{N}$ , i.e., $\forall j\in N_{\geq 0}$ ,

$$y_{j}≔{\left[\begin{array}{cccc} y_{j}\left(1\right) & y_{j}\left(2\right) & \ldots & y_{j}\left(N\right) \end{array}\right]}^{T}$$ (1)

$$\;u_{j}≔{\left[\begin{array}{cccc} u_{j}\left(1\right) & u_{j}\left(2\right) & \ldots & u_{j}\left(N\right) \end{array}\right]}^{T}.$$ (2)

Without loss of generality, the dynamics can be written in the lifted form

$$\forall j\in N_{\geq 0},\;y_{j}=p\left(u_{j}\right),$$ (3)

where ***p*** is the unknown, trial-invariant, nonlinear dynamics. The task consists of updating the input **u**_*j*_ from trial to trial such that the output **y**_*j*_ converges to the desired reference trajectory $r\in R^{N}$ . Tracking performance is measured by the error trajectory

$$\forall j\in N_{\geq 0},\;e_{j}≔r-y_{j}$$ (4)

and root-mean-squared error (RMSE)

$$\forall j\in N,\;e_{j}^{RMS}≔\sqrt{\overset{N}{\underset{i=1}{\sum }}\frac{{\left[e\right]}_{i}^{2}}{N}}.$$ (5)

The problem considered in this work consists in developing a learning method that updates the input trajectory on each trial such that the RMSE decreases. Learning performance is judged based on the progression of the RMSE through trials, and the RMSE shall decline quickly and monotonically. The learning method must not require any a priori model information on the plant dynamics. To support plug and play application, the method must autonomously determine necessary parameters. Furthermore, the method must provide a fair degree of robustness with respect to autonomously determined parameters.

## 3 Proposed Learning Method

**FIGURE 3:**
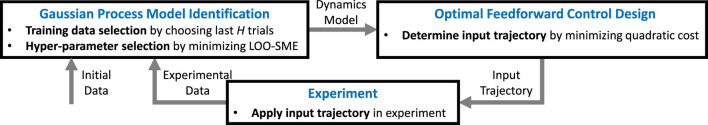
Overview of the proposed learning method: First a Gaussian Process (GP) model is identified, which in turn is used to determine an input trajectory via optimization. The resulting input trajectory is applied in experiment yielding new data to refine the GP model.

We address the proposed problem by an iterative learning scheme, in which each iteration consists of three steps. First, a parameter-free model of the plant dynamics is identified using the experimental data of previous trials, see Section 3.1. To accommodate for possibly nonlinear dynamics, a generic GP model is employed, which predicts the output trajectory for a given input trajectory. Second, the updated input trajectory is determined by solving an optimal feedfoward control problem based on the GP model, see Section 3.2. Third, the updated input trajectory is applied to the plant and resulting data is in turn used to refine the GP model. The structure of the proposed learning scheme is depicted in Figure 3. To enable plug and play application, the proposed method autonomously determines necessary parameters, see Section 3.3.

### 3.1 Gaussian Process Model

We propose a Gaussian Process (GP) model, formally a function $m:R^{N}\mapsto R^{N}$ , that predicts the plant’s output trajectory $\hat{y}\in R^{N}$ based on an input trajectory $u\in R^{N}$ , where the trial index is omitted for sake of notational simplicity.

Let $f\left(v\right):R^{D}\mapsto R$ denote the unknown target function that depends on the regression vector $v\in R^{D}$ . Predictions are based on K∈N observations $z_{k}\in R$ stemming from:

$$\forall k\in \left[1,K\right],\;z_{k}=f\left(v_{k}\right)+w_{k}\;\;\;|\;w_{k}∼N\left(0,\sigma_{w}^{2}\right).$$ (6)

The *K* observation pairs (*z*_*k*_, **v**_*k*_) are collected in the observation training vector $\bar{z}\in R^{K}$ and regression training matrix $\bar{V}\in R^{D\times K}$ , i.e.,

$$\bar{z}≔{\left[\begin{array}{cccc} z_{1} & z_{2} & \ldots & z_{K} \end{array}\right]}^{T},$$ (7)

$$\bar{V}≔\left[\begin{array}{cccc} v_{1} & v_{2} & \ldots & v_{K} \end{array}\right].$$ (8)

The kernel function of two regression vectors $v\in R^{D}$ and $\hat{v}\in R^{D}$ is denoted by $k_{v\hat{v}}\in R$ . The kernel matrix of two regression matrices $V\in R^{D\times K}$ , $\hat{V}\in R^{D\times \hat{K}}$ , which are assembled according to (8), is denoted by $K_{V\hat{V}}\in R^{K\times \hat{K}}$ and has entries ${\left[K_{V\hat{V}}\right]}_{ij}=k_{v_{i}{\hat{v}}_{j}}$ .

Given F∈N test regression vectors assembled in the regression matrix $V\in R^{D\times F}$ , the predicted mean $\mu \in R^{F}$ and covariance $\Sigma \in R^{F\times F}$ are given by:

$$\mu =K_{V\bar{V}}{\left[K_{\bar{V}\bar{V}}+\sigma_{w}^{2}I\right]}^{-1}\bar{z}$$ (9)

$$\Sigma =K_{VV}-K_{V\bar{V}}{\left[K_{\bar{V}\bar{V}}+\sigma_{w}^{2}I\right]}^{-1}K_{\bar{V}V}$$ (10)

The general GP framework can be employed in different ways to model the unknown dynamics (Eq. 3), where the model characteristics are determined by the definition of observation variable *z*, regression vector **v**, and kernel function *k*. First, we exploit the dynamics’ time-invariance by employing a single GP for predicting each output sample. Hence, the observation variable and regression vector are time dependent, i.e., *∀n* ∈ [1, *N*], *z*_*n*_, **v**_*n*_. Now, the model can be chosen to be one of three types, namely finite impulse response (FIR), infinite impulse response (IIR), or state space (SS), which are outlined in the following. A FIR model is obtained when the regression vector consists of the current and all previous input samples, i.e.,

$$\forall n\in \left[1,N\right],\;v_{n}≔{\left[u\left(n\right)\;\ldots \;u\left(1\right)\;\ldots \;0\;\ldots \;0\right]}^{T}.$$ (11)

An IIR model is obtained when the regression vector consists of the current input and the P∈N previous output samples, i.e.,

$$\forall n\in \left[1,N\right],\;v_{n}≔{\left[u\left(n\right)\;y\left(n-1\right)\;\ldots \;y\left(n-P\right)\;0\;\ldots \;0\right]}^{T}.$$ (12)

A SS model is obtained when the regression vector consists of the current input and the previous state sample, which is denoted by $x\in R^{O}$ , i.e.,

$$\forall n\in \left[1,N\right],\;v_{n}≔{\left[u\left(n\right)\;x^{T}\left(n-1\right)\right]}^{T}.$$ (13)

The SS model requires multiple GPs with each predicting the progression of a single state variable (Deisenroth and Rasmussen, 2011b), which not only increases computational complexity, but also requires measurements of the full state vector. Furthermore, IIR and SS models require so called *roll-out predictions*, meaning that the predictions of previous samples are required for predicting the current sample (Deisenroth and Rasmussen, 2011b), and, hence, the matrix $K_{V\bar{V}}$ has to be recomputed for each sample in the output trajectory, which increases the model’s complexity and computational demands. In contrast, the FIR model only requires a single prediction according to Eq. 9 and only a single computation of the matrix $K_{V\bar{V}}$ . We, hence, employ a FIR model, and the regression vector is defined according to Eq. 11 in order to reduce the computational demands of the learning method.

We further choose difference-predictions, i.e.,

$$\forall n\in \left[1,N\right],\;z_{n}≔y\left(n\right)-y\left(n-1\right)\;|\;y\left(-1\right)=0,$$ (14)

which, compared to absolute predictions, increase the model’s capability of extrapolation, see (Deisenroth and Rasmussen, 2011b).

As kernel function, we employ a squared-exponential kernel (SEK)

$$k\left(v,\tilde{v}\right)=\exp\left(-\frac{1}{2l^{2}}{\left(v-\tilde{v}\right)}^{T}\left(v-\tilde{v}\right)\right),$$ (15)

where l∈R is a so called length scale.

Remark 1: SEKs allow a GP to model arbitrary target functions. In the context of dynamic systems, a SEK leads to a nonlinear, time-invariant (NTI) model. Using a squared kernel instead, as e.g.,

$$k_{v\tilde{v}}≔v^{T}\tilde{v},$$ (16)

results in a linear, time-invariant (LTI) model. If the plant dynamics are linear, one may employ a squared kernel to decrease computational complexity in comparison to a NTI model.

To predict an output trajectory $\hat{y}$ for an arbitrary input trajectory **u**, the latter is used to determine *N* regression vectors **v**_*n*_, *n* ∈ [1, *N*], according to Eq. 11, which are assembled in a regression matrix **V** according to Eq. 8. The predicted mean vector ***μ*** follows from Eq. 9. By Eq. 14, ***μ*** contains difference predictions such that the components of $\hat{y}$ follow from the cumulative sum of ***μ***, i.e.

$$\forall n\in \left[1,N\right],\;{\left[\hat{y}\right]}_{n}=\overset{n}{\underset{i=1}{\sum }}{\left[\mu \right]}_{i}.$$ (17)

Mean and covariance predictions require the measurement variance $\sigma_{w}^{2}$ and length-scale *l*, which are so called hyper-parameters. Typically, hyper-parameters are determined based on training data, and numerous approaches have been detailed in the literature (Rasmussen and Williams, 2005). We propose selecting hyper-parameters by minimizing the so called leave-one-out squared-mean-error (LOO-SME). For each of the *k* ∈ [1, *K*] available observations *z*_*k*_, the remaining observation pairs are used to predict *z*_*k*_. The LOO-SME follows from summing the squared difference between the *K* leave-one-out predictions and respective observations *z*_*k*_. Formally, the *k*th LOO-prediction ${\hat{\mu }}_{k}$ is given by

$${\hat{\mu }}_{k}=z_{k}-\frac{{\left[{\left(K_{\bar{V}\bar{V}}+\sigma_{w}^{2}I\right)}^{-1}\bar{z}\right]}_{k}}{{\left[{\left(K_{\bar{V}\bar{V}}+\sigma_{w}^{2}I\right)}^{-1}\right]}_{kk}}$$ (18)

leading to the LOO-SME *e*_LOO_

$$e_{LOO}=\overset{K}{\underset{k=1}{\sum }}{\left(z_{k}-{\hat{\mu }}_{k}\right)}^{2}$$ (19)

and the hyper-parameters $\theta ≔{\left[\sigma_{w}^{2}\;l\right]}^{T}$ follow from

$$\theta =\underset{\tilde{\theta }}{\mathrm{argmin}}\;e_{LOO}.$$ (20)

The optimization problem (Eq. 20) can be solved efficiently, because analytic expressions of the gradients are available, see (Rasmussen and Williams, 2005).

Remark 2: Determining hyper-parameters by LOO-SME minimization is a rather uncommon choice because the variance of the predictions is not taken into account, see (Rasmussen and Williams, 2005). However, we compared LOO-SME minimization with the state-of-the-art method *evidence maximiziation*, as described in Rasmussen and Williams (2005), and we found that performance is superior when using LOO-SME minimization. We assume that this is due to the proposed learning scheme solely relying on the GP’s mean prediction.

GP predictions are known to become computational expensive with increasing amounts of training data (Snelson and Ghahramani, 2006). To overcome this limitation, various data selection approaches have been proposed to reduce training data to a tractable amount, see, e.g., (Seeger et al., 2003; Snelson and Ghahramani, 2006). In the present work, we simply propose limiting the training data to the last H∈N trials.

### 3.2 Optimal Feedforward Control

After the GP model has been identified, it is used to determine an input trajectory that leads to a smaller difference between reference and output trajectory than the input trajectories of previous trials. We propose an optimal control design, where the input is chosen to minimize a quadratic cost criterion. The latter not only considers the predicted tracking error, but also the change of the input trajectory to avoid model inversion and, hence, increase robustness with respect to the uncertainty of the current trial’s model. Formally, the cost criterion is given by

$$\forall j\in N_{\geq 0},\;J\left(u_{j+1}\right)=q{\left\|r-\hat{y}\left(u_{j+1}\right)\right\|}_{2}^{2}+s{\left\|u_{j+1}-u_{j}\right\|}_{2}^{2},$$ (21)

where $q,s\in R_{>0}$ are scalar weights. On each trial, the updated input trajectory **u**_*j*+1_ is chosen to minimize the cost criterion, i.e.,

$$\forall j\in N_{\geq 0},\;u_{j+1}=\underset{\tilde{u}}{\mathrm{argmin}}J\left(u_{j+1}\right)$$ (22)

The optimization problem (Eq. 22) can be solved efficiently since analytic expressions of the cost’s gradient with respect to the input variable can be obtained (Deisenroth and Rasmussen, 2011a).

Remark 3: (Learning to Track Multiple Reference Trajectories)**.** In this paper, we have only considered learning to track a single reference trajectory. This is particularly relevant to applications, in which a robotic system has to repetitively solve a single task, as for example in manufacturing. However, in some applications, a robot has to track a variety of different reference trajectories. In order to accelerate learning in such *multi-reference problems*, the proposed method can be extended to train a GP model based on all previous input/output trajectory pairs, which is then used to determine an optimal initial input trajectory **u**_0_ for any new reference.

### 3.3 Autonomous Parameterization

**FIGURE 4:**
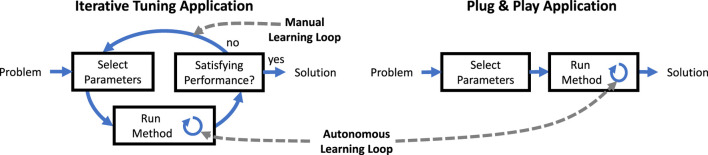
Learning methods typically require some learning parameters. If no procedure for determining the parameters is provided, iterative tuning has to be carried out manually in experiment. If a procedure for determining reliable parameters is available, plug and play application without iterative manual tuning is possible.

Ideally, autonomous learning methods should require neither a priori model information nor manual tuning of parameters. In contrary to previous contributions, the proposed method automatically determines necessary parameters by the procedure outlined in this section, and, as a result, plug and play application is enabled, see Figure 4.

First, we consider the choice of the initial data set I that is used to determine the first GP model and consists of I∈N trajectory pairs (**y**_*i*_, **u**_*i*_), i.e.,

$$I≔\left\{\left(y_{i},u_{i}\right)\;|\;i\in \left[1,I\right]\right\}.$$ (23)

For this purpose, we first determine the largest significant frequency *f*_*O*_ of the reference trajectory. The frequency *f*_*O*_ is used to design a zero-phase low-pass filter ***f***_LP_. The low-pass filter ***f***_LP_ is applied to a zero mean normal distribution with covariance $\sigma_{I}^{2}I$ , and the initial input trajectories are drawn from the resulting distribution, i.e.,

$$\forall i\in \left[1,I\right],\;u_{i}∼f_{LP}\left(N\left(0,\sigma_{I}^{2}I\right)\right).$$ (24)

The input variance $\sigma_{I}^{2}$ is iteratively increased until an input trajectory drawn according to Eq. 24 leads to an output trajectory, whose maximum roughly equals the maximum of the reference, i.e.,

$$u∼f_{LP}\left(N\left(0,\sigma_{I}^{2}I\right)\right)\;\;\;such\;that\;\;\;e_{I}≔{\left\|r\right\|}_{\infty }-{\left\|p\left(u\right)\right\|}_{\infty }\approx 0.$$ (25)

In autonomous parameterization, the number of initial trials is chosen as one, i.e., *I* = 1, to decrease the number of total trials required for the learning. Note that a larger number of initial trials reduces the variance of the convergence speed, i.e., in safety-critical applications a larger number of initial trials may be recommendable. However, using only one initial trial, the proposed method already provides a remarkably safe convergence, as will be demonstrated in Section 4.3. Hence, the autonomous parameterization employs *I* = 1.

Once the parameters *f*_*O*_, $\sigma_{I}^{2}$ , and *I* have been determined, the initial trials are performed, and the weights *q* and *s* are chosen based on the experimental data. The scalar *q*, which weights the error trajectory, is without loss of generality chosen as unity, i.e.,

q=1. (26)

The scalar *s*, which weighs the change in input variable, is chosen as the average squared ratio of output to input maxima over the *I* initial trials, i.e.,

$$s=\frac{1}{I}\overset{I}{\underset{i=1}{\sum }}\frac{{\left\|y_{i}\right\|}_{\infty }^{2}}{{\left\|u_{i}\right\|}_{\infty }^{2}}.$$ (27)

The purpose of the weight selection in Eqs 26–27 is to normalize the cost function (Eq. 21), i.e., we would like the weighted change in the input trajectory to have an impact on the cost function that is equal to impact to the weighted next-trial error trajectory. To achieve this normalization, we employ the squared ratio of the initial trials’ input and output trajectories as described in Eq. 27. Note that the normalization of the cost function only depends on the ratio of the weights *q* and *s* but does *not* depend on their absolute values, hence the choice *q* = 1 is arbitrary and without loss of generality.

The procedure described in this section automatically determines all the necessary parameters without requiring any a priori information on the plant. The following simulations are going to demonstrate that the automatically determined parameters lead to the desired learning performance and that the method provides a fair degree of robustness with respect to the automatically determined parameters.

Remark 4: Note that the proposed autonomous parameterization method aims at successful learning for unknown, nonlinear dynamics and different reference trajectories without requiring any manual adjustment of the parameters. Beyond this aspect, the method may be extended to automatically determine parameters that yield optimal performance in some to-be-defined sense.

## 4 Validation by Simulation

**FIGURE 5:**
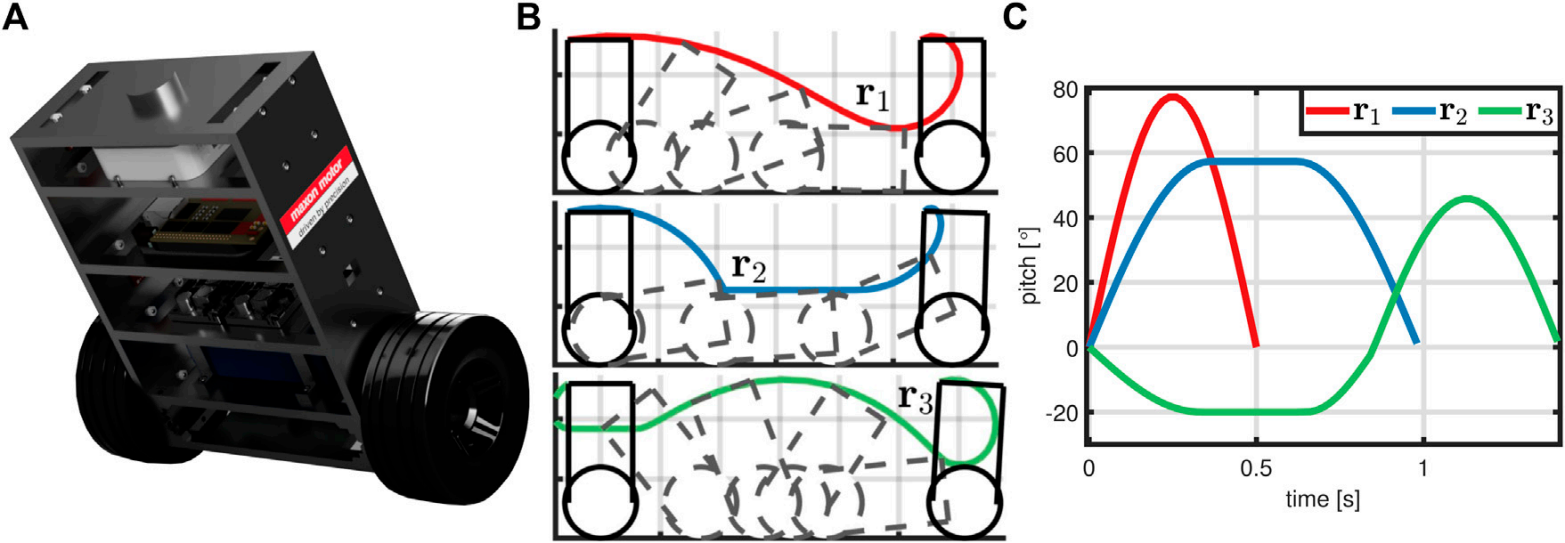
The learning problem: A TWIPR **(A)** is meant to perform three challenging maneuvers **(B)**. The corresponding pitch angle references **(C)** differ in length, amplitude, and frequencies.

In this section, the proposed learning method is validated by simulation of a two-wheeled inverted pendulum robot (TWIPR) that is meant to perform challenging maneuvers, see Figure 5. The TWIPR and automatic determination of learning parameters are presented in Section 4.1. Afterwards, the learning performance for three representative references is investigated in Section 4.2, and the proposed method’s robustness with respect to learning parameters is verified in Section 4.3. Lastly, the effect of the weight *s* on the learning characteristics is studied in Section 4.4.

### 4.1 The Learning Problem

Consider the TWIPR and three desired maneuvers depicted in Figure 5. The corresponding pitch angle reference trajectories are denoted by $r_{1}\in R^{25}$ , $r_{2}\in R^{50}$ , and $r_{3}\in R^{71}$ and formal definitions are given in Supplementary Appendix S1.2. The robot consists of a pendulum body housing main electronics including a microcomputer, inertial measurement units, motors and accumulator. Wheels are mounted onto the motors such that the robot can drive while balancing its chassis. In order to validate the proposed method via simulations, a detailed, nonlinear model of the TWIPR dynamics, see Kim and Kwon, 2015, is implemented. However, the simulation model is completely unknown to the learning method, which can only interact with the simulation by applying an input trajectory and receiving the corresponding output trajectory. Only an approximate, linear model of the dynamics at the upright equilibrium has been obtained, which merely suffices to design a stabilizing feedback controller, see Supplementary Appendix S1.3. Due to the imprecise model, the feedback controller can not track the references precisely, and we instead employ the proposed learning method.

Instead of a state vector, the learning method only requires knowledge of the output variable, which is given by the pitch angle, i.e.,

$$\forall n\in N_{\geq 0},\;y\left(n\right)≔\Theta \left(n\right).$$ (28)

The input variable is given by the motor torque, *∀n* ∈ [1, *N*], $u_{L}\left(n\right)\in R$ .

Application of the proposed learning method requires learning parameters that are automatically determined by the procedure outlined in Section 3.3. We aim at tracking pitch trajectories with a maximum of approximately 75° and spectral content roughly below 5 Hz, i.e.,

$${\left\|r\right\|}_{\infty }\approx 75\;{}^{\circ}\;f_{O}\approx 5\;Hz.$$ (29)

**FIGURE 6:**
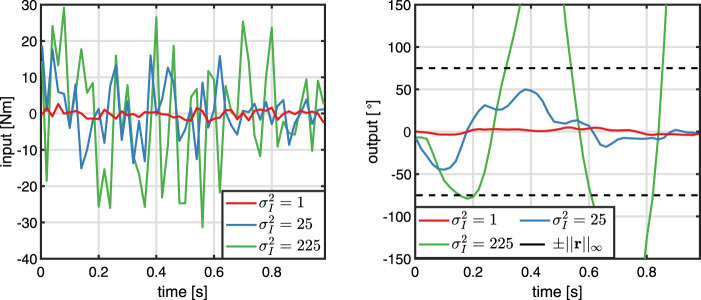
Determination of the input variance $\sigma_{I}^{2}$ : Five different values, of which only three are presented, are used to draw random input trajectories that are applied to the plant. The input variance $\sigma_{I}^{2}=1$ hardly excites the system. In contrary, the input variance $\sigma_{I}^{2}=225$ leads to an output trajectory that significantly exceeds the reference’s maximum. The input variance $\sigma_{I}^{2}=25$ is selected, because the corresponding output trajectory has the same order of magnitude as the reference.

Based on the frequency *f*_O_, a forward-backward, second order Butterworth filter ***f***_LP_ is designed, which is used for drawing initial input trajectories, see Eq. 24. To determine the input variance $\sigma_{I}^{2}$ , the method automatically applies test input trajectories with successively increasing amplitudes to the nonlinear system as described in Section 3.3. A coarse grid $\sigma_{I}^{2}\in \left\{0.005,0.05,1,25,225\right\}$ is chosen, and the algorithm obtains *e*_I_ = [75, 75, 71, 20, −280] and thus selects $\sigma_{I}^{2}=25$ , see Eq. 25 and Figure 6. Note that an even larger and finer grid as well as more sophisticated selection methods could be used, but this simple approach is sufficient because the proposed algorithm exhibits great robustness with respect to the choice of the input variance, as detailed in Section 4.3.

**FIGURE 7:**
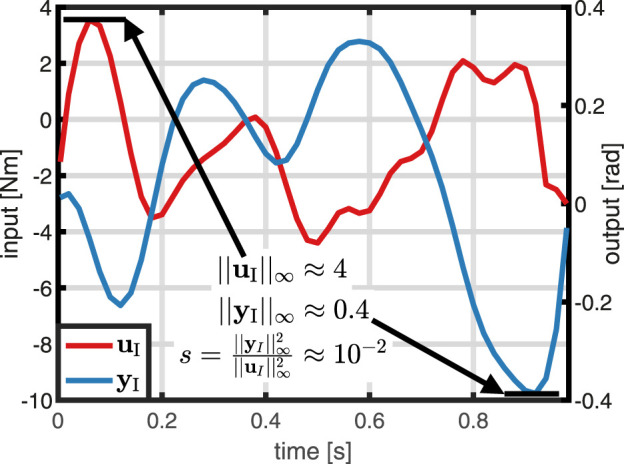
Determination of the weight *s*: Based on the maxima of input and output trajectory in an initial trial, the weight *s* is chosen according to (27).

Next, the weights *s* and *q* of the cost function are determined. According to Eq. 26, *q* = 1 is selected. To determine the weight *s*, one initial trial with the previously determined input variance is performed. As detailed in Figure 7, the value of *s* directly results from Eq. 27 and the experimental data, i.e.,

$$s=\frac{{\left\|y_{I}\right\|}_{\infty }^{2}}{{\left\|u_{I}\right\|}_{\infty }^{2}}\approx \frac{0.4^{2}}{4^{2}}=10^{-2}.$$ (30)

To demonstrate the data-efficiency of the proposed learning method, the training data are limited to the last five trials, i.e., *H* = 5.

### 4.2 Learning Performance

**FIGURE 8:**
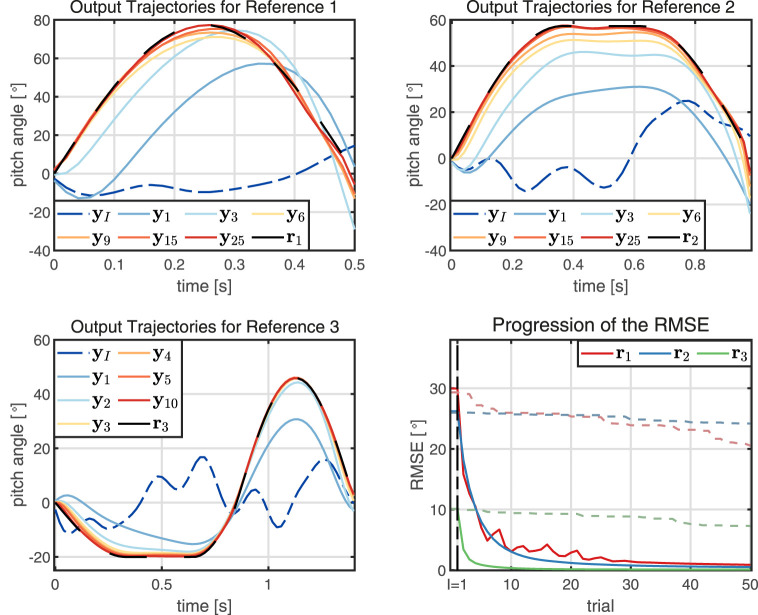
The proposed learning method is employed to track the three desired references. Despite varying lengths, amplitudes and frequencies of the references, satisfying tracking performance is achieved within 10–15 trials. The RMSE is monotonically declining for two of the references and converges to a small value close to zero in all three scenarios. To provide an additional baseline, the dashed lines in the RMSE plot show the performance of a generic reinforcement learning method, which learns magnitudes slower.

First, learning performance for the desired references **r**_1_, **r**_2_, and **r**_3_ is investigated. The parameters are chosen according to the previous section and only one initial trial *I* = 1 is used. In Figure 8, progressions of the output trajectories and error norms over the trials are depicted. For all three references, the proposed method achieves precise tracking after roughly 15 trials. The respective RMSEs rapidly decline over the first trials and converge to a small value close to zero. In case of the references **r**_2_ and **r**_3_, the RMSE decreases monotonically. The simulations demonstrate that, by using the automatically determined parameters, the proposed method rapidly learns to track three different reference trajectories without requiring any system-specific prior knowledge.

Lastly, Figure 8 also depicts the performance of a pure reinforcement learning method, namely a policy-gradient scheme, applied to the same learning task to serve as a baseline for comparison. Details of the policy-gradient algorithm are presented in Supplementary Appendix S1.4 and (Peters and Schaal, 2006). Here, we see that the RMSE declines at a rate that is by magnitudes slower compared to the learning speed of the proposed method. These results are of little surprise because the policy-gradient algorithm is a generic scheme that is not tailored towards the specific task of learning an input trajectory to track a desired reference trajectory. In contrast, the proposed approach leverages the fact that in reference tracking tasks the input/output dynamics of a nonlinear system can be effectively modelled by a FIR GP-model.

### 4.3 Robustness Analysis

The previous simulations have validated the method’s capability of achieving satisfying tracking performance when using the automatically determined parameters. However, as discussed above, presenting results for a single parameterization is of little value. Instead, a learning control method should, ideally, not only achieve satisfying performance for a single parameter configuration, but for a wide parameter space. This is a crucial prerequisite for a method that performs well on different systems for different reference trajectories without any manual adjustments.

To address this question, the proposed method’s robustness with respect to the automatically determined parameters is validated in the following study, where we aim at tracking reference **r**_1_. We consider two different scenarios, namely, the greedy case of one initial trial, *I* = 1, and the conservative case of five initial trials, *I* = 5. Recall the two parameters *s* and $\sigma_{I}^{2}$ , which are the weight in the optimal control problem and the initial input variance.

In the case of *I* = 1, the weight *s* is chosen from the set $S_{1}$ that consists of ten logarithmically spaced values and the initial input variance $\sigma_{I}^{2}$ is chosen from the set $V_{1}$ that consists of ten quadratically spaced values, i.e., for *I* = 1.

$$\;S_{1}=\left\{10^{-1},\ldots ,10^{-3}\right\}\;|S_{1}|=10,$$ (31)

$$\;V_{1}=\left\{3^{2},\ldots ,6^{2}\right\}\;|V|=10,$$ (32)

$$\left(s,\sigma_{I}^{2}\right)\in P_{1}≔S_{1}\times V_{1}\;|P_{1}|=100.$$ (33)

For each of the 100 parameter pairs in $P_{1}$ , 50 *runs* are performed. A *run* *r* consists of choosing a parameter pair ${\left(s,\sigma_{I}^{2}\right)}_{k}$ from $P_{I}$ , producing *I* initial input trajectories, and executing the proposed learning method for an additional 50 trials such that a progression of the RMSE throughout trials is obtained, which we denote by

$$e_{j,k,r}^{\text{RMS}},$$ (34)

where *j* ∈ [0, *I* + *N*] is the trial index, *k* ∈ [1, 100] is the parameter index, and *r* ∈ [1, 50] is the run index.

The same procedure is applied in the case of *I* = 5, but the parameters are chosen from larger sets, i.e., for *I* = 5.

$$\;S_{5}=\left\{10^{-1},\ldots ,10^{-4}\right\}\;|S_{5}|=10,$$ (35)

$$\;V_{5}=\left\{3^{2},\ldots ,7^{2}\right\}\;|V_{5}|=10,$$ (36)

$$\left(s,\sigma_{I}^{2}\right)\in P_{1}≔S_{1}\times V_{5}\;|P_{1}|=100.$$ (37)

**FIGURE 9:**
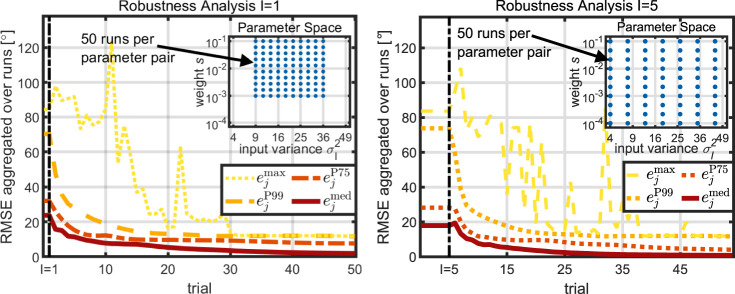
The proposed learning method is run for a total of 5,000 different combinations of parameters and initial data. The RMSE’s maximum over all runs converges to a value significantly lower than the initial. Hence, robust learning is guaranteed for a large parameter space.

To evaluate performance, the maximum $e_{j}^{max}\in R$ , 99th percentile $e_{j}^{P99}\in R$ , 75th percentile $e_{j}^{P75}\in R$ , and median $e_{j}^{med}\in R$ of the RMSE over parameters and runs are considered. Formally,

$$\forall j\in N_{\geq 0},\;e_{j}^{max}≔\underset{k\in \left[1,100\right],r\in \left[1,50\right]}{\max}\left(e_{j,k,r}^{\text{RMSE}}\right),$$ (38)

and $e_{j}^{P99}$ , $e_{j}^{P75}$ , $e_{j}^{med}$ are defined accordingly. Results depicted in Figure 9 show that, for both *I* = 1 and *I* = 5, the maximum of the RMSE converges to a value that is a roughly ten times smaller than the initial value such that the method’s robustness is validated. The RMSE’s 99^th^ percentile is monotonically decreasing, which implies that, besides single outliers, the method achieves the desired form of convergence as defined in Section 2. This means that the proposed method yields desirable performance for a large range of values of the weight *s* and the initial input variance $\sigma_{I}^{2}$ . Furthermore, the RMSE’s median declines below a value of five degrees within 25 trials meaning that satisfying tracking performance is achieved. Lastly, it should be noted that a wider parameter space could be considered in the case of *I* = 5 meaning that, by increasing the amount of initial data, the robustness of the method can be further increased. We, hence, conclude that the simple approach proposed to autonomously determine the weight *s* and the input variance $\sigma_{I}^{2}$ is more than sufficient and can be expected to suffice for more complex systems.

### 4.4 Effects of Weights

The previous analysis has shown that the method rapidly learns to track a desired reference while also being robust with respect to the automatically determined parameters. Next to the use case of automatic plug and play application, the method can also be tuned to meet the needs of a specific application. Hence, we next investigate how the choice of the weight *s* affects learning characteristics, namely the rate of convergence and robustness with respect to initial data. For this purpose, we consider the weights

$$s\in \left\{10^{0},10^{-2},10^{-4},10^{-6}\right\}.$$ (39)

The remaining learning parameters are chosen as one initial trial *I* = 1 and an initial input variance $\sigma_{I}^{2}=25$ . For each weight, 50 runs with differing initial data are performed and performance is judged based on the RMSE’s 90^th^ percentile and median over the 50 runs.

**FIGURE 10:**
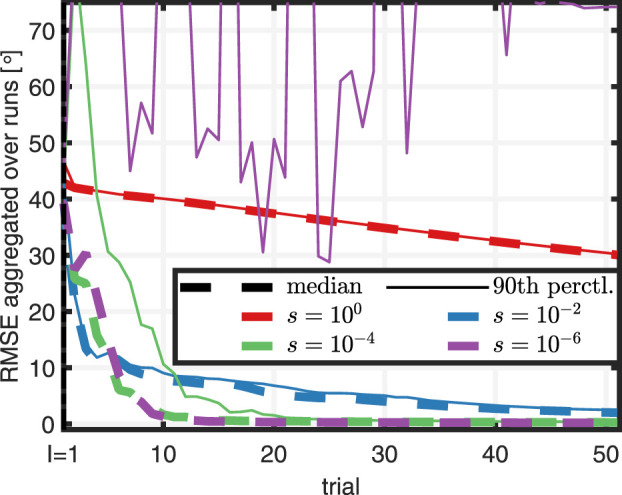
Investigation of the effect of weight *s* on the learning characteristics: Large values of *s* lead to slow learning with small performance variance. Increasing the value leads to faster learning but also a larger variance in performance. Excessively small values of *s* may lead to a RMSE that diverges for some initial data.

Results depicted in Figure 10 show that for a comparatively large value of *s* = 10^0^, the RMSE monotonically declines at a slow pace. Furthermore, there is hardly any difference between median and 90^th^ percentile performance. Decreasing the value to *s* = 10^–2^ leads to a significant increase in speed of convergence. Speed of median convergence can be further increased by lowering the value of the weight to *s* = 10^–4^, which, however, comes at the price of larger 90^th^ percentile RMSEs, which imply an increase in performance variance. If the weight is lowered to an even smaller value, *s* = 10^–6^, median performance is not further increased, but the 90^th^ percentile RMSE does no longer converge meaning that learning fails in a significant portion of runs. In summary, the study indicates that the weight *s* may be used to tune learning behavior, whereby comparatively large values of *s* lead to slow learning that is robust with respect to initial data. Decreasing the value of *s* can increase the speed of learning, but may come at the price of sensitivity with respect to initial data.

## 5 Validation by Experiment

To demonstrate the plug and play applicability of the proposed learning method, it is applied to a real-world TWIPR, which has been previously used to validate learning control methods (Meindl et al., 2020). The robot is meant to dive beneath an obstacle as depicted in Figure 2 with the corresponding reference trajectory $r\in R^{75}$ , *∀n* ∈ [1, 75],

$${\left[r\right]}_{n}=\left\{\begin{array}{cc} 80\sin\left(\pi Tn\right) & n\leq 25\\ 80 & 25<n\leq 50\\ 80\sin\left(\pi T\left(n-25\right)\right) & 50<n \end{array}\right.\left[{}^{\circ}\right].$$ (40)

**FIGURE 11:**
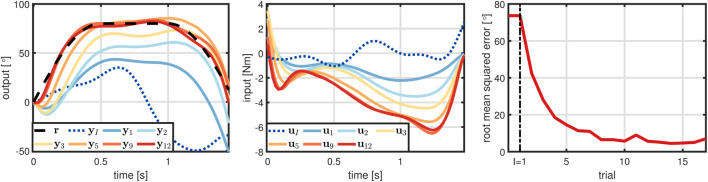
Experimental results of the TWIPR learning to dive beneath an obstacle. Starting from an initial RMSE of roughly 75°, the tracking error rapidly declines over the following trials and sufficiently precise tracking for diving beneath the obstacle is achieved on the seventh trial.

First, the proposed method determines the learning parameters yielding *I* = 1, $\sigma_{I}^{2}=2$ , and *s* = 0.1. The initial input trajectory is drawn according to (Eq. 24) and applied to the TWIPR. The corresponding output trajectory significantly differs from the reference with a RMSE of roughly 75°, see Figure 11. From here onwards, the method iteratively determines a GP model, updates the input trajectory, and performs an experimental trial. Once learning begins, the RMSE rapidly declines, the RMSE drops below 20° on the fourth trial, and a RMSE of less than 10° is reached on the eighth trial. Sufficiently precise tracking precision for diving beneath the obstacle is achieved on the seventh trial and a RMSE close to zero is achieved on the 10th trial. Note, that the RMSE slightly increases on some of the trials, which is likely due to the initial conditions varying from trial to trial.

In summary, the experiments validate that the proposed method enables a real-world robot with unknown, nonlinear dynamics to learn a challenging maneuver. Not only did learning require a small number of trials (≈10) but the method could also be applied in a plug and play manor without iterative tuning of parameters.

## 6 Discussion and Conclusion

In this work, a GP-based learning control scheme has been proposed that autonomously solves reference tracking tasks in systems with unknown, nonlinear dynamics. On each iteration, the unknown dynamics are approximated by a Gaussian Process (GP), which is then used to determine and apply an optimal feedforward control input. The method is completely plug and play, since all required algorithms parameters are determined automatically and manual tuning is avoided. The effectiveness and efficiency of the method were demonstrated by simulations and experiments using the example of a two-wheeled inverted pendulum robot that rapidly learns to perform several challenging maneuvers without any manual tuning or system-specific prior knowledge.

In contrast to previous GP-based learning control approaches, the proposed method overcomes the inherent limitations of time-domain feedback control; it neither assumes knowledge of an effective feedback control structure, nor does it assume the entire state vector to be known. Instead, the proposed method directly adjusts the input based on the measured output. It is therefore as model-agnostic and independent of system-specific prior knowledge as pure reinforcement learning schemes.

While reinforcement learning approaches typically require hundreds of trials for convergence and are therefore unsuitable for experimental validation, the proposed learning control method solves reference tracking problems in a small two-digit number of trials and was successfully validated in real-world experiments.

While the vast majority of previous contributions either validate methods only in simulations or provide only a single results for one carefully chosen parameterization and one specific motion, the present validation has proven effectiveness of the proposed method for several different motions and a large range of algorithm parameterizations. We thereby demonstrated robustness with respect to the automatically determined parameters, and we further investigated the effect of the learning weights on the trade-off between speed of learning and robustness.

We believe that the proposed method is highly suitable for use in kinematic systems that must perform challenging, highly dynamic maneuvers. Beyond the use case of rigid robotics, we expect the proposed method to have a major impact on the development of soft robotics, exoskeletons, and neuroprosthetics, and will therefore contribute to the evolution of autonomous robotic systems that rapidly learn to perform complex, dynamic motions under unknown conditions.

Despite these achievements, the proposed method is subject to remaining limitations. First, only learning to track a single reference trajectory was considered, and future research is going to extend the method to multi-reference tracking tasks. This can either be achieved by the concept outlined in Remark 3 or by combining the proposed method with model-free feeback controller learning to handle trial-varying references and disturbances. Second, the proposed method requires a feasible reference trajectory which might not be directly available in some applications. If the reference is only specified at a subset of the trial’s samples, the proposed method might be extended by well-established *P2P-ILC* concepts, see (Freeman and Tan, 2013; Janssens et al., 2013; Huo et al., 2020). And in cases in which the motion task is formulated only via goal states and constraints, a prior planning step might be required. Third, future research is going to extend the method to be applicable to multi-input/multi-output systems by implementing multiple GP models with each predicting the progression of one of the respective output variables. This may require adjustments like, e.g., sparse GP models, see (Rasmussen and Williams, 2005), in order to reduce the computational demands to a tractable amount. Fourth, the proposed method was validated on one real-world application, and future research will be concerned with applying the method to other, complex real-world applications.

## Data Availability

The raw data supporting the conclusion of this article will be made available by the authors, without undue reservation.
