# Supplementary material for: Bridging Reinforcement Learning and Iterative Learning Control: Autonomous Motion Learning for Unknown, Nonlinear Dynamics
Source: Front Robot AI. 2022 Jul 12;9:793512. doi: 10.3389/frobt.2022.793512 (PMC9315427; doi:10.3389/frobt.2022.793512)
Supplement: Supplementary file 1 [file DataSheet1.pdf]

A Appendices

A.1 Comparison of Feedforward and Feedback Control for Reference Tracking

We consider a discrete-time, linear system of first order with sampling period $T = 0.02$ seconds, output $y \in \mathbb{R}$, and input $u \in \mathbb{R}$. The system is affected by an input delay of one sample leading to state-space dynamics

$$\forall n \in \mathbb{N}, \quad \mathbf{x}(n+1) = \begin{bmatrix} 0.5 & 1 \\ 0 & 0 \end{bmatrix} \mathbf{x}(n) + \begin{bmatrix} 0 \\ 1 \end{bmatrix} u(n)\,, \tag{1}$$

where $\mathbf{x} \in \mathbb{R}^2$ is the state vector. The system's output is affected by measurement noise, i.e.,

$$\forall n \in \mathbb{N}_{\geq 0}, \quad y(n) = \begin{bmatrix} 1 & 0 \end{bmatrix} \mathbf{x}(n) + w(n)\,, \tag{2}$$

where $w(n) \in \mathbb{R}$ is measurement noise that is drawn from a normal distribution with zero-mean and variance 10^{-4}, i.e.,

$$\forall n \in \mathbb{N}_{\geq 0}, \quad w(n) \sim \mathcal{N}(0, 10^{-4})\,. \tag{3}$$

The task consists of having the output y follow the reference $r \in \mathbb{R}$ over a finite horizon of $N = 100$ samples with

$$\forall n \in [1, N], \quad r(n) = \sin(2\pi T n)\,. \tag{4}$$

The feedforward control strategy consists of applying an input trajectory

$$\mathbf{u}_{\mathrm{FF}} := \begin{bmatrix} u_{\mathrm{FF}}(0) & u_{\mathrm{FF}}(1) & \ldots & u_{\mathrm{FF}}(N-1) \end{bmatrix}^T\,. \tag{5}$$

The input values are determined by optimization such that the squared tracking error is minimized, i.e.,

$$\mathbf{u}_{\mathrm{FF}} = \operatorname*{argmin}_{\mathbf{u}} \sum_{n=1}^{N} \left[r(n) - y(n) \right]^2\,. \tag{6}$$

The feedback control strategy consists of a generic, non-linear function to ensure that performance is not limited by the structure of the feedback law. In particular, the input values u_{FB} are computed as the sum of ten polynomials of tenth order, to which the current and nine previous error samples serve as inputs, i.e., $\forall n \in [1, N]$,

$$u_{\mathrm{FB}}(n) = \sum_{i=1}^{10} \sum_{j=1}^{10} k_{ij} \left[r(n) - y(n) \right]^j\,. \tag{7}$$

The set of feedback parameters $\mathcal{K} = \{k_{ij} \mid i, j \in [1, 10]\}$ is determined via optimization such that the squared tracking error is minimized, i.e.,

$$\mathcal{K} = \operatorname*{argmin}_{\tilde{\mathcal{K}}} \sum_{n=1}^{N} \left[r(n) - y(n) \right]^2\,. \tag{8}$$

A.2 Reference Trajectories

The first reference $\mathbf{r}_1 \in \mathbb{R}^{25}$ is given by, $\forall n \in [1, 25]$,

$$[\mathbf{r}_1]_n = 75 \sin(2\pi T n) \,. \tag{9}$$

The second reference $\mathbf{r}_2 \in \mathbb{R}^{50}$ is given by, $\forall n \in [1, 50]$,

$$[\mathbf{r}_2]_n = \begin{cases} 57 \sin(1.38\pi T n) & \forall n \leq 17 \\ 57 & \forall 17 < n \leq 31 \\ 57 \sin(1.38\pi T(n-13)) & \forall 31 < n \end{cases} \; [^\circ] \,. \tag{10}$$

The third reference $\mathbf{r}_3 \in \mathbb{R}^{71}$ is given by, $\forall n \in [1, 71]$,

$$[\mathbf{r}_3]_n = \begin{cases} -20 \sin(1.5\pi T n) & \forall n \leq 16 \\ -20 & \forall 16 < n \leq 31 \\ -20 \sin(1.5\pi T(n-11)) & \forall 31 < n \leq 43 \\ 46 \sin(1.8\pi T(n+13)) & \forall 43 < n \end{cases} \; [^\circ] \,. \tag{11}$$

A.3 Feedback Control of the TWIPR

Consider the dynamics of the TWIPR moving along a straight line. The robot has two degrees of freedom, namely, the pitch angle $\Theta \in \mathbb{R}$ and the position $s \in \mathbb{R}$. The state vector follows with

$$\mathbf{x} = \begin{bmatrix} \Theta & \dot{\Theta} & s & \dot{s} \end{bmatrix}^T \,. \tag{12}$$

The motor torque serves as input variable and is denoted by $u \in \mathbb{R}$. To stabilize the TWIPR in its upright equilibrium, the nonlinear dynamics are approximated by a linear, discrete-time model with state vector $\mathbf{x} \in \mathbb{R}^4$ of the form

$$\forall n \in \mathbb{N}, \quad \mathbf{x}(n+1) = \mathbf{A}\mathbf{x}(n) + \mathbf{B}u(n) \tag{13}$$

using a sampling period of $T = 0.02$ seconds. The stabilizing control input $u_\mathrm{C} \in \mathbb{R}$ is computed by linear state feedback of the form

$$\forall n \in \mathbb{N}, \quad u_\mathrm{C}(n) = -\mathbf{K}\mathbf{x}(n) \,, \tag{14}$$

where the feedback matrix $\mathbf{K}$ is designed by LQR [1].

To track the desired reference maneuvers, the feedback input u_C is superposed by a learned feedforward input u_L leading to the overall input

$$\forall n \in \mathbb{N}, \quad u(n) = u_\mathrm{C}(n) + u_\mathrm{L}(n) \,. \tag{15}$$

A.4 Policy Gradient Implementation

In this section, we briefly outline the implementation details of the finite-difference policy gradient method that was used as a baseline comparison in Section 4.2. For a detailed discussion of the method and its implementation,

see [2]. The finite-difference gradient estimation was chosen because this method is expected to be highly efficient due to the deterministic nature of the simulations, see [2].

In order to apply the policy gradient scheme to the learning task of Section 4.2, the policy is defined as the input trajectory $\mathbf{u}_j$, and the reward of a trial reward is defined as

$$\forall j \in \mathbb{N}_{\geq 0}, \quad R_t := \frac{1}{2}(\mathbf{r} - \mathbf{y}_j)^\top (\mathbf{r} - \mathbf{y}_j)\,. \tag{16}$$

On each iteration, the policy is updated by

$$\forall j \in \mathbb{N}_{\geq 0}, \quad \mathbf{u}_{j+1} = \mathbf{u}_j + \alpha \nabla R_j\,, \tag{17}$$

where ∇R_j is an estimate of the reward's gradient with respect to the input trajectory, and $\alpha \in \mathbb{R}$ is a step-size. To estimate the gradient, $W \in \mathbb{N}$ roll-out trials with the perturbed policies $\mathbf{u}_j + \Delta_w$ are performed, and the gradient is determined by least-squares estimation, as detailed in [2]. In the simulations, the step-size was chosen as $\alpha = 50$, one roll-out per trial, i.e., $W = 1$, was used, and the policy permutations were drawn according to

$$\Delta_w \sim \mathcal{N}(\mathbf{0}, 0.001\mathbf{I})\,. \tag{18}$$

In contrast to the method proposed in this paper, the parameters of the policy gradient scheme had to be tuned manually and were chosen to yield a satisfying trade-off between a fast speed of learning and robust convergence for all three reference trajectories.

References

[1] Frank L Lewis, Draguna Vrabie, and Vassilis L Syrmos. *Optimal control.* John Wiley & Sons, 2012.

[2] Jan Peters and Stefan Schaal. Policy gradient methods for robotics. In *2006 IEEE/RSJ International Conference on Intelligent Robots and Systems.* IEEE, October 2006.
